# Linguistic Markers of Theory of Mind in Spontaneous Speech: A Narrative Review

**DOI:** 10.3390/bs15081016

**Published:** 2025-07-27

**Authors:** Chaimaa El Mouslih, Vegas Hodgins, Lena Palaniyappan, Debra A. Titone

**Affiliations:** 1 Department of Psychology, McGill University, Montreal, QC H3A 0G4, Canada; vegas.hodgins@mail.mcgill.ca (V.H.); lena.palaniyappan@mcgill.ca (L.P.); 2 Montreal Bilingualism Initiative, Montreal, QC, Canada; 3 Center for Research on Brain, Language and Music, McGill University, Montreal, QC H3A 0G4, Canada; 4 Douglas University Mental Health Institute, Department of Psychiatry, McGill University, Montreal, QC H3A 0G4, Canada; 5 Department of Psychiatry, Western University, London, ON N6A 3K7, Canada

**Keywords:** theory of mind, mentalizing, spontaneous speech, speech markers, linguistic indicators, language, cognition

## Abstract

The relationship between theory of mind (ToM) or mentalizing, i.e., the cognitive ability to attribute mental states to oneself and others, and language has been widely explored across disciplines. Identifying reliable linguistic markers of ToM extractable from individuals’ speech provides a promising path for both research and clinical practice. In this narrative review, we aimed to synthesize findings from studies identified through a PSYCINFO search to provide an overview of speech-based markers associated with ToM abilities. Our results revealed six primary categories of relevant speech markers: mental state terms, general linguistic ability, embedded clauses, referring expressions, and pragmatic markers. Standardizing these markers could enhance the replicability and applicability of ToM assessments across diverse populations. We encourage future research to build on these findings to examine how mentalizing is expressed through language in varied social, cultural, and clinical contexts. Advancing this line of inquiry will deepen our understanding of the interplay between language and mentalizing and contribute to broader insights into language and cognition.

## 1. Introduction

“You never really understand a person until you consider things from his point of view… Until you climb into his skin and walk around in it.” (Lee, 1960). As Harper Lee eloquently suggests in this quote from *To Kill A Mockingbird*, true understanding of others goes beyond merely hearing their words—it requires the ability to infer their thoughts, emotions, and perspectives. This cognitive capacity, known as theory of mind (ToM) or mentalizing (often used interchangeably, Frith & Frith, 1999, 2021; Saxe & Kanwisher, 2003), is fundamental to human communication and social interaction. It involves the capacity to attribute mental states, such as beliefs and intentions, to others (Harris, 2017; Premack & Woodruff, 1978). It also encompasses understanding that these mental states can differ from our own, and from reality (Premack & Woodruff, 1978; Wimmer & Perner, 1983), as well as interpreting and predicting others’ behaviors based on these mental states (I. A. Apperly, 2008, 2012; Baron-Cohen et al., 1985). Mentalizing is essential for navigating complex social landscapes, enabling individuals to anticipate and comprehend the behaviors of those around them (Frith & Frith, 2012). In this review, we explore how this capacity is reflected in language use, and how language itself can serve as a window into ToM.

In recent decades, a substantial body of research in developmental psychology has focused on clarifying the timeline and mechanisms underlying ToM development (for review, see Saxe et al., 2004; Slaughter, 2015; Wellman, 2018). A classical method for assessing mentalizing in children is the false-belief task, first introduced by Wimmer and Perner (1983). In this task, the experimenter presents a scenario using toys and props, wherein one character holds a false belief about a situation, and the child must predict the character’s behavior based on that belief. Pivotal studies employing various versions of the false-belief task have consistently demonstrated that theory of mind develops between the ages of three and four, reaching maturity around age five (for a review, see Ensink & Mayes, 2010). This developmental period coincides with significant linguistic growth, suggesting a potential relationship between language development and ToM (for review, see Miller, 2006). Indeed, a substantial body of research highlights the strong connection between these two abilities (Milligan et al., 2007). Throughout the first few years of life, the development of language and theory of mind are interwoven, each undergoing rapid and significant changes that are closely intertwined (Miller, 2006). Language serves as a critical tool for both expressing and understanding mental states, and conversations about thoughts, beliefs, and desires are instrumental in fostering mentalizing abilities in children (Miller, 2006). The use of mental state terms such as think, feel, and know allows children to articulate internal experiences. Similarly, embedded clauses like “she knows that…” help them represent the perspectives and beliefs of others. Furthermore, narrative skills, such as storytelling, enable children to organize and interpret social information, thereby enhancing their mentalizing capabilities (Astington & Olson, 1990).

However, despite the well-established link between theory of mind (ToM) and language, much of the existing research has concentrated on online mentalizing processes—how individuals infer and monitor others’ mental states in real time. This body of work has primarily relied on methodologies such as reaction time tasks (e.g., Dumontheil et al., 2010; Symeonidou et al., 2016), eye-tracking (e.g., Kulke & Hinrichs, 2021; Meijering et al., 2012), standard cognitive tasks (e.g., Navarro & Rossi, 2024; Tiv et al., 2023), neuroimaging techniques (e.g., Koster-Hale & Saxe, 2013; Schurz et al., 2021; Sebastian et al., 2012), and network science (e.g., Navarro et al., 2022; Tiv et al., 2022). In contrast, relatively less attention has been paid to how ToM may be expressed in the content of what people say.

Thus, using language as a means to assess the abstract cognitive skill of mentalizing presents a compelling opportunity. Given the well-established connections between language and ToM (for review, see Miller, 2006), speech characteristics can provide a direct window into underlying mentalizing processes. This approach offers a systematic and direct means of measuring mentalizing abilities and identifying impairments, paving the way for more accessible and accurate assessment methods. Identifying speech variables that reflect ToM in action offers significant advantages for both research and clinical practice.

Indeed, speech-based measurements capture how ToM operates in real time, making them more ecologically valid than traditional tasks like the Sally–Anne test. Standardizing these markers not only facilitates cross-study comparisons but also enhances their reliability, allowing for more consistent and interpretable findings. By improving both standardization and ecological validity, researchers can develop a more nuanced understanding of ToM across diverse populations. This could help resolve ongoing debates, such as whether mentalizing skills differ between bilingual and monolingual individuals—a question that has so far yielded conflicting results (e.g., Navarro & Conway, 2021; S. R. Schroeder, 2018; Sundaray et al., 2018). A standardized approach would ensure that the same variables are systematically examined across studies, reducing inconsistencies in findings. As for clinicians, these assessments offer a more accurate means of identifying ToM deficits as they manifest in real-world interactions, ultimately leading to improved diagnosis and more targeted interventions.

Thus, in this narrative review, we aim to identify a set of speech markers that are both (1) empirically linked to ToM and (2) easily identifiable and extractable for further analysis and prediction. Our methodology focuses on the targeted selection of studies that offer both theoretical and empirical insights into the primary ToM markers that can be derived from speech. Through a selective review of these studies, we aim to provide a broad overview of the speech markers that have been linked to ToM and mentalizing. To identify the relevant literature, we conducted a search of the PSYCINFO OVID database using the following keywords: (Theory of mind OR mentaliz*) AND (Language)*. We then reviewed the search results to select studies that examine the relationship between theory of mind and language and present measurable speech variables linked to mentalizing. This final selection consisted of 20 experimental studies and 2 position pieces, which formed the basis for our narrative synthesis. By synthesizing these findings, we seek to enhance the understanding of the relationship between language and theory of mind across diverse populations.

## 2. Speech Markers of Theory of Mind

Mental states are inherently unobservable. Unlike physical actions, mental processes such as thinking, knowing, liking, and wanting do not have consistent behavioral correlates that can be easily observed or measured. As Gleitman (1990) notes, the meaning of a term like “think” cannot be learned through simple observation, as the associated mental state is not directly visible. Therefore, language serves as a vital source of information about mental states. Without verbal explanations, terms like *guess*, *know*, or *pretend* remain ambiguous. Thus, it is through linguistic input that children gain the necessary context and explanations to understand and use these otherwise invisible states. This linguistic exposure enables them to conceptualize and attribute mental states to themselves and others, supporting the development of ToM.

In this review, we summarize the main speech markers that have been linked to theory of mind in the literature. Table 1 provides an overview of these key markers. Each will be examined in greater detail in the sections that follow. Additional details on the methods, measures and participants of each study reviewed are provided in the Appendix A.

### 2.1. Mental State Terms

One prominent speech marker in the study of theory of mind (ToM) is the use of mental state language, which refers to the frequency and way individuals discuss thoughts, beliefs, desires, and emotions (Astington & Baird, 2005). Numerous studies have demonstrated that children’s exposure to mental state language plays a crucial role in shaping their understanding of mental states. For instance, Ruffman et al. (2002) found that mothers’ use of mental state terms when describing pictures to their 2- to 4-year-old children predicted the children’s later ToM abilities, assessed through a false-belief task. This relationship remained significant even after controlling for factors such as prior ToM skills, language ability, age, and maternal education. Notably, the findings suggest a causal link, as early ToM ability did not predict later increases in mothers’ mental state talk, reinforcing the idea that linguistic input actively facilitates ToM development. In a similar study, Dunn et al. (1991) observed children with siblings at 33 months of age and assessed them 8 months later on false-belief tasks, perspective-taking tasks, and affective labeling tasks, all of which measure ToM skills. Their findings showed that ToM understanding at 40 months was associated with earlier engagement in family discussions about feelings and causality, as well as cooperative interactions with siblings at 33 months.

Building on these findings, several studies (e.g., Jenkins & Astington, 1996; Lewis et al., 1996; Perner et al., 1994; Peterson, 2000) have shown that having siblings provides a developmental advantage in ToM acquisition. This advantage likely stems from the increased opportunities for discourse and social interactions that require understanding others’ thoughts and feelings. More broadly, this reinforces the strong link between language development and ToM, as evidence suggests that greater exposure to mental state discourse enhances children’s ability to reason about others’ minds.

This relationship has been replicated in numerous studies across various populations, further validating the significance of mental state language as an indicator of ToM skills. For example, Grazzani and Ornaghi (2012) conducted a study investigating the relationship between mental state language use and ToM skills in middle childhood. Their findings revealed a moderate correlation between children’s use of mental state terms and their performance on ToM tasks, suggesting that frequent use of such language is linked to a greater ability to understand others’ mental states. Additionally, they found a strong association between children’s comprehension of mental state language and their ToM abilities, highlighting that a deeper understanding of mental state language is strongly associated with advanced ToM skills.

Barreto et al. (2018) explored the relationship between mental state talk and ToM in preschool-aged children, assessing their use of mental state language during a wordless picture book reading task and later measuring their ToM abilities. Their findings revealed a significant association between children’s ToM performance and their use of cognition-related terms, even after controlling for age, verbal ability, and maternal discourse. These findings corroborate previous research (e.g., Ensor & Hughes, 2008; Nielsen & Dissanayake, 2000), suggesting that children who perform better on standardized ToM tasks tend to focus on and refer more frequently to mental states in their discourse. Thus, the quantity of mental state terms produced in spontaneous speech appears to be a strong proxy for ToM abilities.

This pattern has also been observed in atypical populations. For example, Tager-Flusberg (1992) analyzed spontaneous speech in autistic children and age- and language-matched Down syndrome controls, focusing on their references to various mental states. While both groups used similar language when discussing desire, perception, and emotion, autistic children produced significantly fewer references to cognitive mental states and used less language to initiate joint attention. This suggests that while some aspects of mental state talk remain intact in autism, difficulties arise when engaging with others’ thoughts and beliefs. The reduced use of cognitive mental state terms, in contrast to children with Down syndrome, underscores a core ToM deficit in autism, one that affects higher-order mentalizing abilities while sparing references to more concrete mental states. The authors take this as evidence that reduced use of cognitive mental state terms in autism is not simply a byproduct of general cognitive or linguistic delay, but rather a core feature of the condition.

Further supporting the link between mental state language and theory of mind (ToM), a recent neuroimaging study by Paunov et al. (2022) investigated how the brain tracks mental state content in naturalistic stimuli. Using functional MRI, the authors compared the responses of the language network and the ToM network while participants listened to stories that varied in both linguistic and mental state content. They found that the ToM network, which includes regions such as the temporoparietal junction (TPJ) and medial prefrontal cortex (mPFC), selectively tracked the presence of mental state content independently of linguistic complexity. In contrast, the language network was sensitive to linguistic structure but did not selectively respond to mental state information. These findings provide converging neural evidence that the use of mental state terms in speech engages brain regions specifically involved in mentalizing. This strengthens the interpretation of mental state terms as meaningful speech markers of ToM and highlights the value of combining linguistic and neurobiological methods in studying spontaneous mentalizing.

Overall, these studies reinforce the idea that mental state terms serve as a reliable and easily measurable marker of ToM in speech, capturing individuals’ ability to represent and articulate mental states in real-time interactions.

### 2.2. General Linguistic Ability and Vocabulary

Beyond mental state talk, other aspects of language have also been linked to theory of mind. In a longitudinal study, Astington and Jenkins (1999) examined how syntax and semantics contribute to ToM while also assessing their reciprocal relationship. They measured children’s language skills at three time points using the Test of Early Language Development and assessed ToM through false-belief and appearance-reality tasks. Their findings showed that early language abilities, particularly syntax, predicted later ToM performance, even after controlling for prior ToM skills. In contrast, semantics did not play a significant role, and early ToM ability did not predict later language performance. These results underscore the foundational role of early language—especially syntactic competence—in shaping ToM development.

To further illustrate this relationship, Farrar and Maag (2002) conducted a longitudinal study examining how linguistic development at age two predicts ToM performance at age four. They assessed early vocabulary and grammar using the MacArthur Communicative Development Inventory and a naturalistic play session between mother and child. ToM was measured through false-belief, representational change, and appearance–reality tasks, while general language and cognitive development were controlled for using a vocabulary test and a memory-for-sentences test. Their findings reinforced previous research, revealing strong links between early language and later ToM skills. Specifically, a significant association between vocabulary size and ToM scores, as well as a correlation between grammatical complexity (in this study, assessed as parents’ judgements of the morphological complexity of their children’s sentences) at age two and later ToM abilities.

Crucially, the authors also investigated whether this link reflects a general relationship between cognitive and language development or a specific connection between language and ToM. After controlling for general language (Peabody vocabulary scores) and cognitive abilities (memory for sentences), they found that the associations between early language development and later ToM performance remained robust (significant partial correlations between r = 0.46 to 0.83), suggesting a distinct link between these domains. Their findings indicate that general language ability, and particularly vocabulary, is a key predictor of ToM development. This underscores the role of broader language skills, beyond just mental state talk, in shaping children’s ability to understand and attribute mental states. In a similar study, Slade and Ruffman (2005) gave children false-belief and language tasks, designed to tap a different aspect of syntax and semantics. They found that performance on false-belief tasks was predicted by vocabulary size, as well as other syntactic and semantic features of language.

Further supporting this relationship, a large-scale meta-analysis (Milligan et al., 2007) synthesized findings from 104 studies with nearly 9000 children, demonstrating a robust association between general language ability and ToM, independent of age. Language ability accounted for 18% of the variance in false-belief performance, and even after controlling for age, it explained 10% of the variance. Notably, all aspects of language—including syntax, semantics, receptive vocabulary, general language ability, and memory for complements—were significantly related to false-belief understanding, with memory for complements showing the strongest effect (44% of the variance), followed by syntax (29%) and semantics (23%). These findings offer strong support for the role of semantics in ToM development, in contrast to earlier studies such as Astington and Jenkins (1999). Such inconsistencies may reflect methodological differences in how language and ToM constructs were measured across studies. The findings suggest that while vocabulary knowledge plays a role in ToM development, broader linguistic competencies, particularly syntactic complexity and memory for sentence structures, may be even more critical.

Building on this work, van Dijk et al. (2023) further examined the relationship between language and ToM by using both vocabulary size and general syntactic proficiency as proxies for mentalizing abilities in children’s freely told narratives. They applied a feature-based natural language processing (NLP) approach to analyze these narratives and extract features relevant to theory of mind. Using linguistic features such as lexical complexity, syntactic structure (e.g., dependency distance, clausal complementation), pragmatic markers, and social word use, they trained a logistic regression model to predict the mental depth of story characters as a proxy for ToM. The classifier achieved a macro F1 score of 0.71 on an initial test set and showed stable performance across 100 different train–test splits, though external validation on an independent dataset was not conducted. Their results indicated that vocabulary size, general syntactic proficiency, and pragmatic markers were associated with greater character depth in the narratives, suggesting a stronger capacity for theory of mind.

Taken together, these findings suggest that various aspects of language—particularly syntactic complexity, vocabulary size, and memory for complements—are closely linked to theory of mind and may serve as potential proxies in research. Given their consistent associations with false-belief understanding, these linguistic markers could be very valuable in assessing mentalizing across different populations.

### 2.3. Embedded Clauses

Another feature of speech linked to mentalizing abilities is the understanding and use of embedded clauses (de Villiers & Pyers, 2002). Embedded clauses are subordinate clauses that provide additional information within a sentence, indicative of complex syntactic structures and advanced cognitive processing (de Villiers & Pyers, 2002). For example, in the sentence *“She believes that he will come to the party”*, the clause *“that he will come to the party”* is an embedded clause that adds depth to the main clause *“She believes”.*

The use of embedded clauses, particularly those encoding mental state attributions (e.g., *she thinks that… or he believes that …)* is associated with theory of mind (ToM) abilities, as they reflect an individual’s capacity to process and convey complex thoughts about their own and others’ mental states (de Villiers & Pyers, 2002). The ability to produce and comprehend such sentences indicates advanced linguistic and cognitive skills, including the capacity to attribute mental states and intentions. This is particularly evident when the embedded statements are false (e.g., “*She thinks that the sky is red*”), as producing and understanding these statements requires the speaker to adopt another’s perspective and transcend their own knowledge of reality. The use of false embedded clauses demonstrates a speaker’s ability to consider and articulate beliefs or knowledge that diverge from their own, thereby showcasing their ToM capabilities.

de Villiers and Pyers (2002) conducted a longitudinal study examining the relationship between sentence complements (a kind of embedded clauses) comprehension and false-belief reasoning. They assessed children aged 3–5 years four times over the course of a year, using tasks that measured both false-belief understanding and mastery of sentence complements. Their results revealed that mastery of sentence complements predicted later success on false-belief tasks, but not vice versa. Their findings thus suggest that grasping this syntactic structure not only precedes but may be a prerequisite for successful false-belief reasoning.

Zhang et al. (2023) investigated the use of embedded clauses in Mandarin-speaking individuals with psychosis, analyzing spontaneous speech from patients with schizophrenia and healthy controls. To assess theory of mind (ToM), participants completed the verbal Animated Triangles Task (Abell et al., 2000), which measures intentionality attribution, and the non-verbal Brüne’s Picture Sequencing Task (Brüne, 2003), which evaluates false-belief understanding at multiple levels. The results showed a significant correlation between the production of embedded argument clauses and ToM performance across both tasks, whereas associations between linguistic measures and general neurocognitive abilities were weaker and less consistent. This suggests that the use of complex syntactic structures in speech is closely linked to mentalizing skills, highlighting embedded clauses as a potential linguistic marker of ToM abilities.

However, it is important to note that Wiltschko (2024) responded to this study, raising concerns about the linguistic criteria used to identify embedded clauses in spontaneous speech. Specifically, the author argues that certain constructions, such as the Mandarin *ba* construction, were classified as embedded argument clauses without sufficient linguistic justification. As a result, they may not actually reflect ToM-related syntactic complexity, and their inclusion may compromise the validity of the marker. This calls into question whether the observed correlation truly captures the relationship between embedded clauses and mentalizing or whether it is influenced by alternative linguistic or cognitive factors. Therefore, caution is warranted when interpreting these findings.

Additionally, this link between embedded clauses and mentalizing has also been observed in autism spectrum disorder (ASD). K. Schroeder et al. (2021) assessed children’s performance on metarepresentational tasks measuring ToM alongside their understanding of embedded clauses. Their findings revealed that deficits in false belief and intentionality—both key components of mentalizing—were predicted by difficulties in embedded clause comprehension. These results further support the link between understanding and using embedded clauses and the ability to reason about mental states.

Beyond simple correlational studies, some researchers have investigated the relationship between theory of mind and embedded clauses through training studies, which can demonstrate specific causal relationships. Lohmann and Tomasello (2003), for example, investigated whether linguistic training could enhance false-belief understanding in three-year-old children. The study exposed children to deceptive objects—items that appear to be one thing but are actually another (e.g., a flower that is actually a pen)—and engaged them in discussions using different sentence structures (embedded or simple clauses). The findings revealed that mere exposure to deceptive objects did not improve children’s false-belief understanding. However, training with embedded clauses alone was sufficient to enhance false-belief reasoning, even in the absence of deceptive objects, underscoring the central role of language in ToM development. This represents a significant contribution, as previous studies typically combined deceptive objects with embedded clauses, making it unclear whether improvements stemmed from the deceptive nature of the objects or the linguistic structure. By isolating sentence structure, this study provides clear evidence that embedded clauses independently facilitate false-belief understanding, suggesting a causal role of language in ToM development. These findings support de Villiers’ hypothesis that embedded clauses offer children a helpful, if not necessary, representational format for conceptualizing and discussing false beliefs.

Building on this work, training studies offer a promising avenue for developing interventions aimed at improving theory of mind (ToM) skills, particularly in populations where ToM deficits are pronounced, such as autism spectrum disorder (ASD). Durrleman et al. (2023) explored this potential by training children with ASD and neurotypical peers on embedded clauses. Their findings revealed that the training enhanced ToM performance across groups, and that these improvements were maintained 4 to 6 weeks after training. Additionally, they showed that participants with milder ASD symptoms showed the most significant gains. These findings suggest that training on embedded clauses can be an effective tool for strengthening ToM abilities in children with ASD, particularly those with milder symptoms.

Overall, we have highlighted the link between the production and comprehension of embedded clauses and ToM across various populations, including clinical samples such as those with ASD and schizophrenia. This connection underscores the importance of developing standardized markers for identifying ToM deficits in specific disorders, like ASD and schizophrenia. While much of the evidence is correlational, training studies provide stronger support for a causal relationship by demonstrating that targeted exposure to embedded clauses can enhance false-belief reasoning. One proposed mechanism is that embedded clauses offer a linguistic structure for explicitly representing beliefs that diverge from reality, a core function of ToM (de Villiers & Pyers, 2002).

### 2.4. Referring Forms

Another aspect of spontaneous speech linked to theory of mind (ToM) is the use of referring forms. These include definite articles (e.g., *the*), indefinite articles (e.g., *a*, *an*), demonstrative determiners (e.g., *this*, *that*, *these*, *those*), demonstrative pronouns (e.g., *this* one, *those* ones), and personal pronouns (e.g., *he*, *she*, *they*) (Gundel et al., 2007). The link between these linguistic elements and ToM lies in the cognitive processes required to use and understand them effectively.

Gundel and Johnson (2013) conducted a corpus study to examine children’s use of referring expressions in spontaneous discourse and its implications for ToM development. Their findings indicated that by age 3, children were already using referring expressions appropriately, preceding the emergence of ToM abilities at age 4. They interpret this as evidence that mastering these expressions marks an initial step toward mentalizing. Specifically, they argue that the appropriate use of referring expressions is inherently tied to mentalizing, as it requires speakers to assess their interlocutor’s memory and attentional focus regarding the intended referent. Using determiners and pronouns correctly demands that speakers consider the listener’s knowledge. For instance, referring to “the book” suggests that the speaker assumes the listener is familiar with the object in question, requiring the speaker to construct a mental model of the listener’s knowledge—a fundamental aspect of ToM. Similarly, determiners such as “this” and pronouns like “it” depend on the listener’s ability to interpret contextual cues, reinforcing the necessity of understanding what aspects of the environment are salient to the listener. Gundel et al. (1993) offer a theoretical account of this process, proposing that determiners (e.g., *the*, *this*, *that*) and pronouns (e.g., *it*, *this*, *that*) encode, as part of their conventional meaning, the cognitive status of the referent for the listener. That is, they signal how accessible or activated the referent is in the listener’s memory and attention at the point just before the expression is encountered. Thus, the speaker must assess and predict the listener’s cognitive state and adjust their language accordingly. For example, using “it” assumes the referent is already in the listener’s focus of attention. Effective use of these linguistic elements also involves making inferences about the listener’s intentions and knowledge, such as using “this” to draw attention to something new or “that” to refer to something known.

In another study comparing clinical samples with typically developing children, Lorusso et al. (2007) examined the presence and quality of theory of mind (ToM) indicators in the narrative production of children with different genetic syndromes associated with ToM impairments and distinct patterns of linguistic strengths and weaknesses. The study included children with Down syndrome, Cornelia de Lange syndrome, and Williams syndrome, alongside typically developing peers. Participants’ speech was recorded, and various linguistic variables thought to reflect ToM were extracted, such as perceptual verbs (e.g., *see*, *look*, *hear*), personal pronouns presupposing reference to known characters, and emotional verbs (e.g., *cry*, *shout*). The results indicated that children with intellectual impairments performed differently from typically developing children on narrative tasks, despite being matched on mental age. This finding suggests that the emergence of ToM is closely tied to linguistic development, reinforcing the connection between ToM and language skills. More importantly, and particularly relevant to our argument, among all linguistic indicators of ToM, personal pronouns emerged as the most sensitive in differentiating the clinical groups from typically developing individuals. Specifically, individuals with intellectual impairments not only used personal pronouns less frequently but also omitted them more often where they were necessary for effective communication. This pattern of misuse reflects difficulties in the pragmatic rather than syntactic use of pronouns, which in turn reflects mentalizing difficulties.

In summary, the use of determiners and pronouns appears closely linked to theory of mind (ToM), as it requires speakers to infer and accommodate their listeners’ mental states, reflecting their mentalizing abilities. As such, the frequency and appropriateness of referring expressions in spontaneous speech could serve as an indirect indicator of mentalizing skills. Atypical use—whether infrequent or inappropriate—may signal potential ToM difficulties. To better understand this relationship, further experimental studies are needed to explore how determiners and pronouns in spontaneous speech align with explicit ToM measures across different populations. This remains a compelling direction for future research.

### 2.5. Pragmatic Markers

Rubio-Fernandez (2021) presents a compelling argument in their position piece, focusing on two specific pragmatic markers: demonstratives (e.g., this vs. that) and articles (e.g., a vs. the). In this context, pragmatic markers refer to words that signal the speaker’s assumptions about the listener’s cognitive state, especially what is known, new, or shared in the discourse. While demonstratives and articles are also considered referring expressions, the author here emphasizes their pragmatic function due to their role in establishing joint attention and signaling shared knowledge (Rubio-Fernández et al., 2019; Tomasello, 2008). Expanding on this connection, the author argues that language plays a fundamental role in the development of ToM, challenging the notion that ToM is purely an innate capacity (e.g., I. Apperly, 2011). Pragmatic markers, the author contends, facilitate perspective-taking and the understanding of others’ mental states, both core components of ToM (Baron-Cohen, 1995; Rubio-Fernández et al., 2019).

Rubio-Fernandez underscores the importance of studying language and ToM together to fully grasp their interdependent development. The author introduces a novel perspective, suggesting that these abilities may have co-evolved and co-developed through the acquisition and use of pragmatic markers (Rubio-Fernandez, 2021). While this hypothesis offers a compelling theoretical framework, it is important to acknowledge that empirical support for the co-development of language and ToM via pragmatic markers remains limited. Nevertheless, this view aligns with our research objective of examining ToM through speech markers. By analyzing speech as a window into mentalizing, we can move beyond treating language and ToM as separate skills and instead explore the intricate ways in which they shape and reinforce one another.

To build on this perspective, van Dijk et al. (2023) empirically investigated the relationship between pragmatic markers and theory of mind competence. As described earlier in this paper, they analyzed children’s freely told narratives and extracted various linguistic features using natural language processing (NLP), including pragmatic markers, and examined their association with character complexity, which served as a proxy for higher ToM. Pragmatic markers were operationalized following Rubio-Fernandez (2021) as words that signal deixis and common ground, including demonstratives (e.g., *this*, *that*, *here*, *there*) and the definite article (*the*). Their findings revealed that narratives featuring more complex characters (greater “character depth”) also contained more pragmatic markers, reinforcing Rubio-Fernandez’s position and further supporting the idea that language and ToM are deeply interconnected.

While this section has focused on morphosyntactic elements such as articles and demonstratives as pragmatic markers linked to theory of mind, it is worth noting that broader pragmatic abilities (e.g., understanding indirect speech acts, metaphors, irony, or implied meaning) also rely heavily on perspective-taking and mental state attribution (see special issue on pragmatics, Buchanan et al., 2021). Indeed, recent reviews emphasize how pragmatic comprehension and ToM overlap substantially at the cognitive level (Frank, 2018). These forms of inference may also manifest in spontaneous speech. Future work could explore whether such phenomena can be operationalized as speech markers of theory of mind, particularly in ecologically valid contexts, to expand the current scope of measurable linguistic indicators of mentalizing.

### 2.6. Definitional Competence

Another promising yet relatively novel speech marker appears to be *definitional competence*, or the ability to produce explicit, accurate definitions of words. This ability is metalinguistic by nature and reflects metarepresentational thinking, making it conceptually relevant to ToM. Although it remains understudied, recent work has begun to explore its connection to ToM. For instance, Bianco et al. (2022) examined a sample of older adults and found that ToM performance significantly predicted definitional competence performance. Their findings suggest that both abilities rely on shared cognitive processes involved in reflective language use and perspective-taking.

Similarly, Cornaggia et al. (2024) investigated adolescents and showed that individuals who were better able to provide abstract, relational, and socially contextualized definitions also demonstrated stronger ToM reasoning. Importantly, their study differentiated between crystallized lexical knowledge and more nuanced metalinguistic awareness, suggesting that definitional tasks tap into deeper cognitive–linguistic integration. While the evidence is still preliminary, these findings point to definitional competence as a potentially valuable marker of ToM ability, particularly because it can be elicited through structured yet open-ended speech tasks. Future research should further investigate this link across developmental and clinical populations to clarify its diagnostic utility and theoretical implications.

## 3. Future Directions

In this narrative review, we have provided an overview of specific speech markers that reflect ToM abilities, such as mental state talk, vocabulary size, and embedded clauses, as summarized in Table 1 (with methodological details of each study in Appendix A). These markers are valuable for studying ToM as they help deepen our understanding of its development and manifestation across different populations. As highlighted in the introduction, it would be informative to use such markers to resolve ongoing debates. For instance, it would be valuable to compare monolingual and bilingual populations and compare their ToM skills. Indeed, while developmental studies consistently show an advantage for bilingual children in ToM development (for a review, see S. R. Schroeder, 2018), research on adults has produced conflicting results.

For instance, Rubio-Fernández and Glucksberg (2012) conducted a study comparing bilingual and monolingual adults using a traditional false-belief task paired with eye-tracking. They found that, while all adults exhibited an egocentric bias when reasoning about others’ beliefs, bilinguals were less prone to this bias than monolinguals. Furthermore, performance on the false-belief task correlated significantly with executive control, suggesting that stronger ToM abilities are linked to better executive functioning. The authors interpret these findings as evidence that bilinguals’ enhanced executive control and sociolinguistic sensitivity may explain their advantage in ToM and false-belief reasoning. Several other studies have corroborated this bilingual advantage in ToM (Berguno & Bowler, 2004; Goetz, 2003; Gordon, 2016; Kovács, 2009, 2011; Navarro & Conway, 2021; Nguyen & Astington, 2014), raising questions about the mechanisms underlying this advantage.

However, some studies have not found a bilingual advantage. Sundaray et al. (2018) tested younger and older adults on pragmatic inference tasks and reported no significant differences between monolinguals and bilinguals. Additionally, Antoniou (2022) examined irony interpretation (a form of mentalizing) in multilingual, bilingual, and monolingual young adults, finding no group differences in either accuracy or processing speed. These findings suggest that certain aspects of mentalizing may not differ between multilingual and monolingual individuals.

These inconsistent findings may stem, in part, from a lack of control over key individual differences in bilingual populations, such as language proficiency and patterns of language use, all of which can influence theory of mind (ToM) performance (Białecka et al., 2024; Tiv et al., 2021). Indeed, bilingualism is heterogeneous: balanced versus dominant language profiles, daily versus context-specific code-switching, and culturally bound language practices each shape cognitive outcomes. Indices such as language entropy capture this day-to-day diversity and predict pragmatic skills beyond the age of acquisition or total exposure (Gullifer & Titone, 2020). Second-language experience likewise modulates first-language irony comprehension, another ToM-relevant skill, underscoring the need to model bilingual sub-types when assessing ToM (Tiv et al., 2021). Without systematic control over bilingual characteristics, it remains difficult to isolate the effects of bilingualism on ToM.

In addition, studies vary widely in the types of tasks used to assess ToM, ranging from traditional false-belief tests (e.g., unexpected location and unexpected contents) to non-traditional tests (e.g., visual–spatial perspective) (S. R. Schroeder, 2018). This variability in task design likely contributes to the mixed results observed in the literature, making it difficult to isolate the effects of bilingualism on ToM.

Thus, establishing a standardized set of markers to measure ToM sets the stage for meaningful comparisons across diverse populations and provides a clearer framework for understanding the variability in ToM skills across languages and contexts. Indeed, developing a standardized, and potentially automated, approach to measure ToM, to supplement the diverse explicit tasks currently used in the literature (e.g., false-belief tasks, appearance–reality tasks, and representational change tasks), could be highly valuable. Such a method would provide a more direct and concrete way to assess an abstract skill, minimizing the confounding factors often present in behavioral tasks. In doing so, it could help explain the inconsistencies reported in previous research. This approach would offer significant benefits for research, such as unifying methods across studies, facilitating replication, and improving our understanding of ToM across diverse populations.

This approach also holds significant potential when applied to clinical populations with active theory of mind (ToM) impairments. Theory of mind impairments occur in various disorders, such as schizophrenia (for a review, see Brüne, 2005) and autism spectrum disorder (for a review, see Baron-Cohen, 2000). These deficits significantly affect social functioning, leading to challenges in social communication which impact relationships and overall quality of life. Addressing these deficits necessitates effective interventions based on a comprehensive understanding of one’s ToM abilities. Such an understanding enables the development of strategies tailored to the complex needs of individuals with these impairments. However, it is important to keep in mind that computational methods, such as NLP, come with their own set of challenges, including dealing with informal language or words that have multiple meanings (Khurana et al., 2023), as well as concerns with the ecological validity of speech samples, when collected using constrained elicitation tasks (Mackinley et al., 2021).

Considering this, there is an urgent need to develop robust and practical methods for assessing ToM abilities, particularly in contexts where direct questioning is not feasible. Indeed, traditional ToM assessments often rely on explicit tasks that require verbal responses (e.g., Sally–Anne task, Wimmer & Perner, 1983). However, these tasks can be particularly challenging for individuals with severe cognitive or psychological impairments, young children, or simply uncooperative adult patients. For instance, during episodes of active psychosis, individuals may be unable to engage with direct questions about false beliefs or other ToM-related constructs, leading to high attrition rates (Farris et al., 2020). However, subtle aspects of their speech, such as pronoun usage, referential coherence, or the complexity of sentence structure, may reveal insights into their mental state (de Boer et al., 2020). Thus, supplementing traditional explicit tasks with these linguistic markers, which could serve as proxies for ToM functioning, would enable clinicians to assess impairments without the need for traditional, controlled testing environments. However, their clinical use still requires systematic validation to ensure they are not used in inappropriate contexts or interpreted inaccurately.

In addition to diagnostic utility, these standardized measures could play a key role in the development of targeted interventions. Understanding the specific nature of ToM deficits in clinical populations can help design interventions that are tailored to the cognitive and social challenges faced by individuals with conditions such as schizophrenia. For example, deficits in mental state attribution could be directly addressed by interventions that enhance social cognition, ultimately improving patients’ ability to navigate complex social situations. Additionally, establishing clear, standardized ToM metrics allows for more precise comparisons between neurotypical individuals and those with clinical conditions, facilitating a deeper understanding of how ToM deficits manifest in clinical populations. Such precision is crucial not only for improving diagnostic accuracy but also for refining our understanding of ToM-related impairments across various disorders.

Moreover, tracking these ToM metrics over time offers a valuable tool for assessing the efficacy of interventions. By monitoring changes in ToM-related speech markers, clinicians can obtain real-time feedback on how well an intervention is working, allowing for timely adjustments and personalized treatment approaches. Beyond intervention efficacy, these markers also provide a means to track the progression of disorders, potentially offering insight into their course over time. In conditions like schizophrenia, where ToM deficits are often dynamic, such markers could even serve as early indicators of relapse, allowing clinicians to intervene more swiftly (Zaher et al., 2024). Thus, integrating ToM speech markers into clinical practice not only enhances our understanding of ToM impairments but also opens new avenues for diagnosis, treatment, and long-term management of clinical populations. This approach holds the potential to transform how we conceptualize and treat mentalizing deficits across various mental health disorders.

However, it is important to highlight that the studies reviewed lack attention to contextual variability in eliciting speech. They do not account for how different communicative settings—such as social conversations versus structured activities like storytelling or roleplaying—may differentially elicit mental state talk. Indeed, since storytelling and roleplay encourage discussions of third-person mental processes and require the establishment of common ground, they may naturally promote more frequent and complex ToM-related language. In contrast, spontaneous dialogue may elicit less mental state language, depending on the topic and interpersonal dynamics. Future research should explore how study design and task selection influence observed patterns of mental state talk, for example, by directly comparing narrative tasks (e.g., storytelling) with more open-ended, conversational speech elicitation paradigms. Additionally, linguistic alignment, which is the tendency for speakers to unconsciously adapt their prosody, word choices, and syntax to match their interlocutors (Pickering & Garrod, 2013), has been largely overlooked in studies examining ToM-related language. This phenomenon, central to models such as Pickering and Garrod’s Interactive Alignment Model (IAM) (Pickering & Garrod, 2013), suggests that speakers adjust their language based on their conversational partner’s expectations and prior discourse patterns, potentially shaping the use of mental state language. Investigating this alignment through structured dialogues or analyses of turn-taking patterns could shed light on its role in ToM expression. The influence of linguistic alignment on linguistic markers of ToM, especially in populations that have been shown to have impaired linguistic impairments, like schizophrenia (Dwyer et al., 2020), remains an open question, warranting further investigation. Finally, all studies reviewed were conducted in English. Future research should examine whether the relationships between the reviewed speech markers and ToM hold across languages, as cross-linguistic investigations are essential for improving the generalizability and applicability of results. Addressing these gaps will provide a more nuanced understanding of how ToM processes manifest in natural discourse.

Taken together, incorporating speech markers of mentalizing, as summarized in this review (see Table 1), into future studies and potentially clinical practice emerges as a highly valuable endeavor. Additionally, it remains essential to continue investigating other potential speech markers that may not yet have been identified or are still understudied (e.g., definitional competence), as these could further enrich our understanding of mentalizing and its clinical applications.

## 4. Conclusions

In summary, this review has underscored the central role of theory of mind (ToM) in human interactions, demonstrating its essential function in enabling individuals to understand and interpret the mental states of others. When mentalizing is impaired, as observed in conditions such as autism spectrum disorder (ASD) and schizophrenia, significant challenges arise in social communication and perspective-taking, ultimately affecting an individual’s quality of life. This review has also highlighted the intricate relationship between language and ToM, showing that language functions both as a tool for and a reflection of mentalizing abilities. Linguistic elements such as mental state talk, embedded clauses, vocabulary size, and pragmatic markers provide valuable insights into the cognitive mechanisms underlying ToM, offering concrete, quantifiable measures of this abstract skill. Identifying these markers is especially relevant for clinical populations, where traditional ToM tasks may be difficult to administer or interpret reliably. While these speech markers show promise, their empirical and clinical validity must still be confirmed through comparative and longitudinal studies before they can be reliably used in applied settings.

The use of language-based measures to assess mentalizing has important implications for both research and clinical practice. Though these methods require further standardization and validation, speech markers present a naturalistic and ecologically valid means of evaluating ToM, allowing researchers and clinicians to study cognitive functioning in real-world contexts. These markers not only facilitate ToM research in typical populations but also hold potential for identifying and addressing ToM deficits in clinical populations. The findings discussed in this review highlight the promise of language-based markers as both diagnostic tools and intervention targets. By leveraging these markers, future research can refine our understanding of ToM, particularly in individuals with developmental or psychiatric conditions.

To advance this field, we encourage further research on older participants, extending beyond childhood studies, aligning with the call raised by I. A. Apperly et al. (2009). Indeed, most of the studies reviewed focus on early childhood, yet the relationship between language and ToM may evolve across development, with different patterns potentially emerging in adolescence and adulthood. Additionally, more studies are needed on clinical populations to determine the applicability of speech markers in diagnosing and tracking mentalizing impairments. In particular, comparing these markers across clinical and nonclinical populations could help determine their sensitivity and reliability as clinical markers of mentalizing deficits. We also advocate for increased use of natural language processing (NLP) methods, as demonstrated by van Dijk et al. (2023), to automate and enhance the analysis of these markers. NLP techniques offer a powerful means of systematically extracting and quantifying linguistic features from speech, reducing the need for time-intensive manual coding. By leveraging machine learning and computational modeling, these methods can identify subtle patterns in speech that may be indicative of mentalizing abilities, allowing for more precise and scalable assessments. Integrating NLP into mentalizing research could facilitate large-scale studies, improve diagnostic accuracy in clinical populations, and provide objective, replicable measures for tracking mentalizing abilities over time. That said, practical limitations remain, including the need for large, annotated clinical datasets to train robust models, and the risk of model bias when algorithms are applied across diverse populations with varying speech profiles.

In conclusion, we urge future researchers to use the speech markers identified in this review (see Table 1) to further investigate ToM across diverse populations and contexts, particularly through longitudinal designs, naturalistic interaction studies, and validation in clinical populations. Expanding this line of inquiry will contribute to a deeper understanding of mentalizing and its linguistic underpinnings, ultimately paving the way for innovative tools in both diagnosis and intervention.

## Figures and Tables

**Table 1:** Speech Markers of Theory of Mind (ToM).

Type of Marker	Description	Population Studied	Key Findings
***Mental State Terms***	Words that describe internal mental processes such as thoughts, feelings, desires, perceptions, or intentions (e.g., *think*, *wish*, *pretend*, *hate*).	Children: between 53 and 60 months (Barreto et al., 2018); between 8 and 10 years old (Grazzani & Ornaghi, 2012); with autism, between 3 and 7 years old (Tager-Flusberg, 1992).	Exposure to and use of mental state terms improves ToM ability (e.g., Barreto et al., 2018; Grazzani & Ornaghi, 2012). Deficits noted in autism (Tager-Flusberg, 1992).
***General Linguistic Ability***	Broader language skills such as syntax, vocabulary, and memory for complements.	Children; 3 years old (Astington & Jenkins, 1999); 2 years old (Farrar & Maag, 2002); 4 to 12 years old (van Dijk et al., 2023); under age 7 (Milligan et al., 2007).	Early language abilities (esp. syntax and vocabulary size) linked to later ToM performance (Astington & Jenkins, 1999; Farrar & Maag, 2002) and are proxies for assessing ToM development (Milligan et al., 2007; van Dijk et al., 2023).
***Embedded Clauses***	Complex sentence structures embedding one proposition within another (e.g., *She thinks that he is lying).*	Children; 3 to 5 years old (de Villiers & Pyers, 2002); 3 years old (Lohmann & Tomasello, 2003); 8 years old (Durrleman et al., 2023).	Understanding and using embedded clauses is a prerequisite for and is linked to ToM (de Villiers & Pyers, 2002). Training with embedded clauses enhances false-belief reasoning (Lohmann & Tomasello, 2003) and shown effective in ASD interventions (Durrleman et al., 2023).
***Referring Forms***	Use of definite articles (e.g., *the*), indefinite articles (e.g., *a*, *an*), demonstrative determiners (e.g., *this*, *that*, *these*, *those*), and pronouns (e.g., *he*, *she*, *it*, *they*, *we*).	Children; between 1 and 9 years old (Gundel & Johnson, 2013); 6 and 14 years old (Lorusso et al., 2007).	Proxy for mentalizing abilities (Gundel & Johnson, 2013). Inappropriate use in conditions with ToM deficits (Lorusso et al., 2007).
***Pragmatic Markers***	Contextual linguistic elements like demonstratives (e.g., *this* vs. *that*) and articles (e.g., *a* vs. *the*).	Children; 4 to 12 years old (van Dijk et al., 2023).	Likely indicators of ToM competence (Rubio-Fernandez, 2021); linked to character complexity in narratives (van Dijk et al., 2023).
***Definitional Competence***	Ability to produce explicit, accurate definitions of words.	Adults, 21 to 85 years old (Bianco et al., 2022); adolescents, 14 to 19 years old (Cornaggia et al., 2024).	Performance on definitional competence tasks is linked to better ToM performance (Bianco et al., 2022; Cornaggia et al., 2024).

## Data Availability

Not applicable.

## Appendix A. Summary of Study Characteristics

**Citation**	**Paper Type(s)**	**Aim**	**Population**	**Place of Origin**	**Measures**	**Method**
Ruffman et al. (2002)	Quantitative Original Research	Investigates whether mothers’ use of mental state language predicts children’s later theory of mind understanding, beyond children’s own mental language, age, language ability, and SES.	n = 82 children (ages 2–5) and their mothers. White middle and upper-middle class, rural and urban UK areas.	United Kingdom	CELF language test (Linguistic Concept subtest at all time points) Theory of mind composite (false belief, desire–emotion, desire–action, emotion–situation [Time 1 only], justification, ambiguity, wicked desires [Time 3 only]) Picture task (mental state utterances: desire, emotion, cognition [think/know], modulation of assertion) Maternal education (7-point SES scale)	Longitudinal study with 3 time points over 1 year; picture description tasks, transcribed utterances coded; theory of mind tasks and standardized language tests administered.
Dunn et al. (1991)	Quantitative Original Research	Examines individual differences in young children’s understanding of others’ emotions and beliefs and identifies early family-related predictors.	n = 50 children (mean age = 33 months at T1, 40 months at T2), their mothers, and older siblings; families from central Pennsylvania.	United States	Affective labeling task (identify emotions in facial expressions) Affective perspective-taking task (infer others’ emotions from scenarios) False-belief task (standard unexpected-location task) Conversational coding (feeling state talk, causal talk) Mean length of utterance (MLU) (child language complexity) Parent education (years of schooling) Occupational SES (parent job status) Family interaction rating scales (sensitivity, responsiveness, affective climate)	Longitudinal study with two home visits (at 33 and 40 months); observational recordings and rating scales at T1, standardized sociocognitive tasks at T2 (affective labeling, perspective-taking, false belief); discourse transcribed and coded; multiple regression analyses.
Grazzani and Ornaghi (2012)	Quantitative Original Research	Investigates the relationships between children’s use and comprehension of mental state language and their theory of mind abilities.	n = 110 children (mean age = 9 years 7 months; aged 8–10), balanced by gender; recruited from Italian primary schools.	Italy	TAM-2 language test (verbal ability—metalinguistic awareness) Theory of mind tasks (second-order false-belief stories) Test of Emotion Comprehension (TEC) (belief-based and mixed emotions) Mental state verb comprehension task (definition selection for psychological verbs) Describe-a-friend task (use of mental state terms: emotion, cognition, volition)	Cross-sectional design; standardized tasks and written responses; measures administered individually; data analyzed using correlations and multiple regression.
Barreto et al. (2018)	Quantitative Original Research	Examines the association between ToM and mental state talk at 55 months and their relationship with social competence and behavior at 69 months.	n = 73 preschool children (mean age = 55 months) and their mothers.	Portugal	Theory of mind tasks (Diverse Beliefs, Knowledge Access, Unexpected Contents I & II, Explicit False Belief, Unexpected Location) Picture book task (mother and child mental state terms: desire, cognition, emotion) Peabody Picture Vocabulary Test-Revised Social Competence and Behaviour Evaluation Scale (teacher report: aggression, anxiety–withdrawal, competence)	Longitudinal design with 2 time points (T1 = 55 months, T2 = 69 months); lab-based interaction and assessments at T1; teacher report at T2; controlled for maternal education, age, verbosity, and child gender
Tager-Flusberg (1992)	Quantitative Original Research	Investigates whether autistic children show deficits in the early acquisition of mental state language (desire, perception, emotion, cognition) compared to children with Down syndrome as an index of theory of mind development.	n = 12 children (6 autistic boys; 4 boys with Down syndrome, 2 girls with Down syndrome), matched on age and language level; families from various SES levels.	United States	SALT language transcript system (coded for utterances referencing desire, perception, emotion, cognition) Functional coding of mental state terms (e.g., conversational use vs. true mental state reference) Elaboration coding (causality, contrast with reality) Self/Other reference coding Leiter International Performance Scale (non-verbal IQ) Mean Length of Utterance (MLU) Index of Productive Syntax (IPSyn) Lexical Diversity (number of different word roots per 100 utterances)	Longitudinal study over 1–2 years; bimonthly home visits; 40–70 min recorded parent–child play sessions; transcripts coded using SALT system; utterances analyzed for psychological state terms and functions.
Astington and Jenkins (1999)	Quantitative Original Research	Examines whether language competence predicts later theory of mind development or vice versa and whether syntax or semantics contribute differentially to this relation.	n = 59 children (29 girls, 30 boys), aged 2 years 9 months to 3 years 10 months at start; all from middle-class English-speaking homes; 4 spoke additional languages.	Canada	Test of Early Language Development (assesses general language ability, with separate subscores for syntax and semantics, both expressive and receptive) Theory of mind composite score (based on performance on three tasks: change-in-location false-belief task, unexpected-contents false-belief task, and appearance–reality task) Internal consistency reliability coefficients reported for both language and theory of mind measures at all time points	Longitudinal design with 3 testing points across 7 months; children assessed at beginning, middle, and end of nursery school year; hierarchical regression analyses used to examine predictive relations between language and theory of mind over time.
Farrar and Maag (2002)	Quantitative Original Research	Examines whether children’s language abilities at 2 years of age (vocabulary and grammar) predict their theory of mind performance at 4 years of age.	n = 20 children (9 girls, 11 boys); tested at 24–27 months, and again at 4–5 years (mean age = 52 months).	United States	MacArthur Communicative Development Inventory (MCDI) (parent-reported vocabulary size and grammatical complexity at 24 and 27 months) Mean Length of Utterance (MLU) (derived from naturalistic mother-child play sessions at 27 months, analyzed via CHILDES/CLAN system) Theory of mind tasks (assessing appearance-reality, false belief, and representational change; adapted from Gopnik & Astington, Welch-Ross) Peabody Picture Vocabulary Test (PPVT-R) (general vocabulary ability at 4 years) Woodcock–Johnson Memory for Sentences (verbal memory at 4 years)	Longitudinal design: toddlers tested at 24 and 27 months (language), followed up at 4 years for theory of mind tasks; multiple correlational and partial correlation analyses conducted to assess associations between early language and later theory of mind.
Slade and Ruffman (2005)	Quantitative Original Research	Examines longitudinal bidirectional relations between syntax, semantics, working memory, and theory of mind in preschool children. Tests whether syntax uniquely contributes to theory of mind development and whether working memory mediates the relation.	n = 44 preschool children (25 boys, 19 girls), mean age 3.8 years at Time 1; all native English speakers from 4 nurseries.	United Kingdom	Semantics: British Picture Vocabulary Scale Semantics: Linguistic Concepts subtest Syntax: Word Order Test (adapted from Caramazza & Zurif, 1976) Syntax: Embedded Clause Test (adapted from Ruffman et al., 2002) Theory of mind composite (two unexpected-transfer false-belief tasks and one unexpected-contents task) Working Memory task: modified Backwards Digit Span (adapted from Davis & Pratt, 1995)	Longitudinal design with two time points, 6 months apart; testing conducted individually at nursery; hierarchical regressions and cross-lagged analyses used to examine predictive relations; sensitivity analyses conducted with subsampling procedures.
Milligan et al. (2007)	Meta-analysis	Quantifies the strength of the relation between language ability and false-belief understanding in children under 7 years old; examines moderators such as type of language ability assessed, type of false-belief task, and direction of effect.	Meta-analysis of 104 studies; total n = 8891 children under 7 years old.	Multiple countries including United States, Canada, and United Kingdom	Language measures: receptive vocabulary (e.g., PPVT), general language, syntax, semantics (varies across studies) False-belief measures: standard false-belief tasks (unexpected transfer, unexpected contents, etc.) Moderator variables coded: age, type of language measure, type of false-belief task, direction of effect, clinical vs. non-clinical samples	Meta-analytic methods: effect sizes computed from correlations; moderator analyses conducted; controlled for age; cross-lagged effect sizes analyzed for directionality of effect.
van Dijk et al. (2023)	Quantitative Original Research	Investigates the relation between language ability and theory of mind in children’s freely told narratives using natural language processing (NLP) tools and classification models.	n = 442 children (ages 4–12), recruited from primary schools, daycares, and community centers.	Netherlands	Character Depth (CD) coding system (labels stories into Actor, Agent, or Person levels reflecting mental state complexity) Lexical Complexity (LC) (calculated using perplexity based on lemma frequencies from the BasiScript corpus) Lexical Diversity (LD) (measured via Measure of Textual Lexical Diversity [MTLD]) Dependency Distance (DD) (syntactic complexity; mean dependency length via spaCy parsing) Clausal Complementation (CC) (number of sentential complements per utterance) Pragmatic Markers (PM) (use of deixis and definite articles as perspective indicators) Social Words (SOC) (LIWC social category word counts) Lemmas (binarized bag-of-words representations for classification)	Recorded live storytelling sessions during workshops; expert-annotated narratives for CD level; feature extraction using NLP tools; logistic regression classifier; model interpretation via Shapley values; 80/20 train-test split; robustness tested over 100 resampling splits.
de Villiers and Pyers (2002)	Quantitative Original Research	Examines whether mastery of complex syntax, especially sentential complements, predicts children’s false-belief understanding over time.	n = 28 children (13 boys, 15 girls), aged 3–5 years old at the start of the study (range 3–1 to 3–10 years), recruited from local preschool and daycare centers.	United States	Memory for Complements task (elicitation of complement structures with mental verbs [think/believe] and communication verbs [say/tell], 12 items total) Spontaneous Speech Samples (analyzed using Index of Productive Syntax [IPSyn]: overall IPSyn, complements, complex sentences, sentence scores) Mean Length of Utterance (MLU) Medial Wh-question task (long-distance wh-questions testing complement understanding) False-belief tasks: – Unexpected Contents (Perner et al., 1987) – Unseen Displacement (Wimmer & Perner, 1983) – Explanation of Action (Bartsch & Wellman, 1989)	Longitudinal study over 1 year with 4 testing periods; individual child assessments at daycare/preschool centers; multiple regression and pass/fail contingency analyses conducted to examine directionality between language and theory of mind measures.
Zhang et al. (2023)	Quantitative Original Research	Investigates relations between syntactic complexity in Mandarin Chinese and theory of mind performance in schizophrenia patients versus healthy controls.	n = 90 total participants (51 schizophrenia patients, 39 healthy controls); all native Mandarin speakers of Han Chinese ethnicity.	Multiple countries including China, Denmark, Spain and United States	Animated Triangles Task (Abell et al., 2000) (used both for spontaneous speech elicitation and ToM scoring) Brüne’s Picture Sequencing Task (Brüne, 2003) (non-verbal ToM measure with 1st-, 2nd-, and 3rd-order false-belief questions) Brief Assessment of Cognition in Schizophrenia (BACS) (assesses verbal memory, working memory, motor speed, verbal fluency, attention, executive function) Linguistic Annotations (via CLAN system): – Embedded Argument Clauses – Embedded Adjunct Clauses – Aspect Markers (Progressive: zai, zhengzai; Completeness: le, guo) – Non-clausal Adjuncts (V-attached, VP-attached, Epistemic Adverbs) Clinical Scales: SAPS, SANS, PANSS (for symptoms)	Participants completed narrative descriptions during the Animated Triangles Task; speech was recorded and manually annotated; mixed-effects negative binomial regression models and Spearman correlations used to analyze relations between linguistic features, ToM, neurocognition, and clinical symptoms.
Wiltschko (2024)	Position Piece/Review	Provides a linguistic critique of Zhang et al. (2023), arguing that their classification of embedded argument clauses in Mandarin is theoretically problematic and challenges their conclusions about syntax and theory of mind in schizophrenia.	N/A	Spain	N/A	Theoretical analysis of linguistic classifications used in Zhang et al. (2023); reviews linguistic literature on Mandarin ba-construction, argument/adjunct clause distinctions, and mental content representation; evaluates implications for ToM research in schizophrenia.
K. Schroeder et al. (2021)	Quantitative Original Research	Investigates how intensionality, theory of mind (ToM), and complex syntax relate in children with autism spectrum conditions (ASCs) compared to typically developing (TD) peers and whether understanding embedded clauses predicts performance on metarepresentational tasks.	n = 50 children (25 ASC, 25 TD), mean age ≈ 9 years. All Catalan–Spanish bilinguals from Spain. Children were matched on VMA. ASC diagnoses confirmed via ICD-10 and ADOS. No intellectual disability in ASC group.	Spain	Sally–Anne Task (false-belief/true-belief understanding) Intensionality Task (dual-description reasoning with appearance-reality distinctions) Relative Clause Comprehension (e.g., “Show me the boy who hugs the girl”) Complement Clause Comprehension, subdivided into: – Says that (e.g., interpreting true/false statements) – Sees that (factive complements) – Seems that (appearance-based judgments) Peabody Picture Vocabulary Test (PPVT-III) for VMA	Experimental design with two sessions (~25 min each) using picture selection tasks delivered via PowerPoint. Tasks assessed metarepresentational and syntactic abilities using matched visual formats across conditions. Participants’ responses analyzed using logistic mixed-effects regression.
Lohmann and Tomasello (2003)	Quantitative Original Research	Examines whether different types of linguistic experience (specifically sentential complement syntax and perspective-shifting discourse) causally contribute to the development of false-belief understanding in young children.	n = 138 German children (aged 3 years 3 months to 3 years 10 months), from various SESs, all native German speakers. All participants had typical language development and no demonstration of false-belief understanding at pre-test.	Germany	Vocabulary Pretest (Kaufman Assessment Battery for Children) False Belief Pretest and Post-tests – Representational Change Task – Appearance–Reality Task – Change-in-Location Task – Sentential Complement Pre-tests and Post-tests – Tom Test/Memory for Complements – Reported Speech Tasks	Children were randomly assigned to one of four training groups: full training (discourse + syntax), discourse-only, sentential-complement-only, or no-language. Each received three sessions over two weeks in their preschool. Training involved structured interactions using deceptive objects or stories, depending on group. Standardized pretests and post-tests assessed false-belief and complement syntax understanding.
Durrleman et al. (2023)	Quantitative Original Research	Investigates whether training on sentential complements (e.g., “X says that…”) can improve ToM in children with autism spectrum disorder and to examine which individual characteristics predict training success.	n = 53 children, 33 with ASD (ages 5 years 7 months to 14 years 9 months) and 20 neurotypical (ages 3 years to 6 years). All were native French speakers.	Switzer-land and France	Verbal False-Belief Task (e.g., adapted Sally–Anne scenario) Low-Verbal False-Belief Task (minimizing linguistic demands) Diverse Desire and Belief Tasks (ToM precursors) Complements Comprehension Test (e.g., “Dad says that…”) Raven’s Coloured Progressive Matrices (RPM) Exalang 3–6 Scale (language and cognitive skills) Childhood Autism Rating Scale (CARS) (for ASD group)	Children completed pre-, post-, and delayed (4–6 weeks later) assessments. Training on sentential complements (especially communication verbs) was delivered via the DIRE iPad app over 4–6 weeks (8–12 sessions). Tasks used animated scenarios followed by selection or repetition activities targeting syntactic structures relevant to ToM.
Gundel and Johnson (2013)	Case Study	Examines young children’s use of referring expressions (e.g., pronouns, articles, demonstratives) within the Givenness Hierarchy framework and explores how these usages reflect early-developing components of theory of mind.	Analyzed naturalistic speech data from 9 English-speaking children (aged 1 year 9 months to 2 years 8 months) from the CHILDES database. Data drawn from the Valian, Brown, and Bloom corpora. All were typically developing children observed in their home, daycare, or playroom.	United States	Spontaneous Speech Samples from CHILDES corpora Use of Referring Expressions, categorized as – Personal pronouns (e.g., he, she, it) – Demonstrative pronouns (e.g., this, that) Demonstrative determiners (e.g., this N, that N) – Definite articles (the N) – Indefinite articles (a N)	Corpus analysis of children’s spontaneous utterances coded for referring expressions and the cognitive status (e.g., in focus, activated, familiar) of the referents, based on the Givenness Hierarchy framework. Each referring form was analyzed for whether it matched the assumed mental state of the addressee, using established coding guidelines. Inter-coder reliability was 94%.
Lorusso et al. (2007)	Case Study/Quantitative Original Research	Investigates how different linguistic profiles in individuals with genetic syndromes affect ToM indicators in narrative production and evaluates whether ToM development is more closely tied to language or cognitive development.	n = 42 individuals (6 with Cornelia de Lange syndrome, 6 with Williams syndrome, 6 with Down syndrome, and 24 typically developed). All participants were native Italian speakers.	Netherlands and Italy	Narrative Production Task using a 6-frame picture story ToM-related Indicators, including: – Direct/Indirect Speech – Psychological, Perceptual, and Declarative Verbs – Personal Pronoun Usage – Shifts in Point of View – Linguistic Links (e.g., “then,” “and”) Cognitive Assessments – Wechsler Intelligence Scales for Full Scale and Mental Age – Fabbro’s Battery and Test di Valutazione del Linguaggio (TVL) for a subset (CdLS group only)	Participants were asked to tell a story based on a picture sequence. Narratives were audio-recorded, transcribed, and analyzed for ToM-related language features. Statistical comparisons and correlations examined the influence of cognitive and linguistic factors across groups.
Rubio-Fernandez (2021)	Position Piece/Review	Proposes that pragmatic markers (especially demonstratives and definite articles) form a critical link between language and ToM, enabling their co-development and co-evolution via a positive feedback loop across three timescales: language acquisition, use, and historical change.	N/A	United States	Reviews studies using the following: False-Belief Tasks (e.g., Sally–Anne task) Demonstrative Comprehension Tasks (e.g., “this” vs. “that” across contexts) Article Usage Tasks (e.g., definite vs. indefinite noun phrase production) Narrative and Discourse Analyses Cross-linguistic and Typological Comparisons Corpus and Developmental Data (e.g., CHILDES)	The author reviews cross-linguistic, psycholinguistic, and developmental research to support the hypothesis that pragmatic markers scaffold early ToM abilities like joint attention and perspective-taking, and that their evolution parallels ToM development across cultures and history.
Cornaggia et al. (2024)	Quantitative Original Research	Explores the relationship between two metarepresentational abilities in adolescence (ToM and definitional competence) while also examining the effects of age and gender on these abilities.	n = 75 Italian adolescents (age range: 14–19 years). Participants attended three types of public high schools (lyceum, technical, and professional) in central Italy. All native Italian speakers, no neurodevelopmental or psychiatric disorders.	Italy	Reading the Mind in the Eyes Test (RMET) – Assesses inference of complex mental states from eye-region photos. Co.De. Scale (Definitional Competence) – Evaluates quality of definitions for 32 words (nouns, adjectives, verbs, emotion terms), scored from 0–6. – Subscores: ○ ToM Words ○ Non-ToM Words ○ Emotions ○ Non-emotional Mental States	Participants completed both tasks in a single 1-h classroom session. Data were analyzed using *t*-tests, correlations, and hierarchical regressions to assess the effects of age, gender, and the predictive relationships between ToM (RMET) and definitional competence.
Bianco et al. (2022)	Quantitative Original Research	Investigates how ageing affects two metarepresentational abilities (ToM and definitional competence) and whether ToM predicts definitional skill beyond the effects of age and education.	n = 74 participants (24 aged 21–55 years old, 25 aged 60–70 years old, and 25 aged 71–85 years old.	Italy	Reading the Mind in the Eyes Test (Adult version) Co.De. Scale (Definitional Competence): – 32 target words (ToM vs. non-ToM) scored on a 7-level scale – Assesses semantic, syntactic, and metalinguistic complexity	Participants completed the Eyes Task and Co.De. Scale in a 1 h session at home. Scores were analyzed using ANOVAs, correlations, and hierarchical regressions to examine effects of age, education, and ToM on definitional ability.
Paunov et al. (2022)	Quantitative Original Research	Examines whether language and ToM brain networks differentially track linguistic and mental state content in naturalistic stories.	n = 40 adults (ages 19–45, mean = 26 years)	United States	fMRI, naturalistic story listening, annotations of linguistic vs. mental state content	Participants completed fMRI while listening to naturalistic stories. Statistical analyses used mixed-effects linear regression to compare tracking of linguistic and mental state content across language and ToM regions of interest (ROIs).
